# Endoscopic submucosal dissection for superficial esophageal squamous cell carcinoma in patients with cirrhosis and esophageal varices

**DOI:** 10.1002/deo2.117

**Published:** 2022-05-24

**Authors:** Tomoaki Mochimaru, Takuto Hikichi, Minami Hashimoto, Jun Nakamura, Mika Takasumi, Tsunetaka Kato, Ryoichiro Kobashi, Takumi Yanagita, Rei Suzuki, Mitsuru Sugimoto, Yuki Sato, Hiroki Irie, Tadayuki Takagi, Masao Kobayakawa, Hiromasa Ohira

**Affiliations:** ^1^ Department of Gastroenterology School of Medicine Fukushima Medical University Fukushima Japan; ^2^ Department of Endoscopy Fukushima Medical University Hospital Fukushima Japan; ^3^ Medical Research Center Fukushima Medical University Fukushima Japan

**Keywords:** endoscopic submucosal dissection, esophageal cancer, esophageal varices, liver cirrhosis, squamous cell carcinoma

## Abstract

Endoscopic submucosal dissection (ESD) has become the standard treatment for superficial esophageal squamous cell carcinoma (SESCC). However, the treatment strategy for SESCC complicated by esophageal varices (EVs) has not been established. We report two cases of SESCC in patients with alcoholic cirrhosis complicated by EVs who underwent ESD. Case 1 presented with EVs on the anal side of the SESCC, and endoscopic variceal ligation (EVL) was performed before ESD. After EVL, the SESCC was successfully treated by ESD without any adverse events. Case 2 presented EVs from the anal side of the SESCC to the submucosa just below the SESCC. Then, EVL and endoscopic injection sclerotherapy with polidocanol were performed before ESD. However, ESD was not completed because of severe bleeding by uncontrolled blood flow below and around the SESCC. Bleeding during ESD was controlled in case 1, but not in case 2.

## INTRODUCTION

Endoscopic submucosal dissection (ESD) has become the standard treatment for superficial esophageal squamous cell carcinoma (SESCC) with a low risk of lymph node metastasis.^1^ On the other hand, Esophageal varices (EVs) are important complications in patients with liver cirrhosis (LC),^2^ and EV bleeding is a cause of fatal outcomes. In addition, SESCC and EVs may simultaneously occur in the LC patient with heavy alcohol drinkers. If ESD for SESCCs is performed first, the risk of bleeding from EVs may become a severe issue during ESD. If endoscopic treatment of EVs is performed first, the risk of perforation during ESD may increase because of fibrosis after EV treatment. However, only a small number of cases have reported the treatment of SESCC complicated with EVs,^3^, ^4^, ^5^, ^6^, ^7^, ^8^, ^9^ and the treatment strategy has not been established yet. Herein, we describe the clinical courses of SESCC in patients with alcoholic LC complicated by EVs who underwent ESD.

## CASE REPORT

**FIGURE 1:**
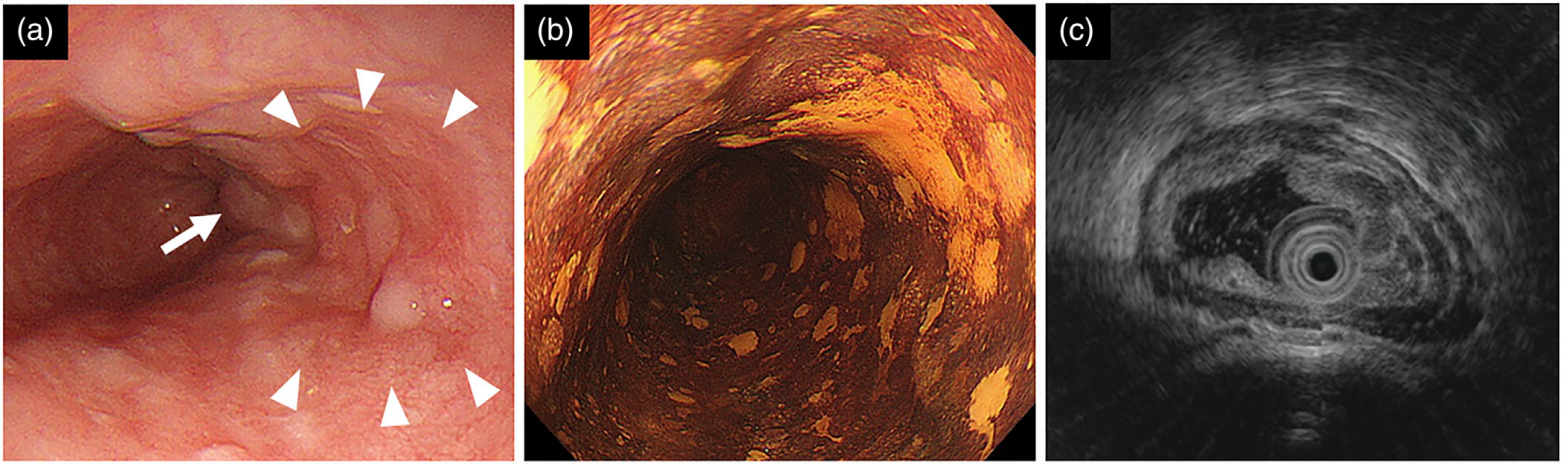
Endoscopic findings before treatment in case 1. (a) A 15‐mm flat and reddish squamous cell carcinoma was found on the right wall of the middle thoracic esophagus (arrowhead). An esophageal varix was also observed in the anal side of the squamous cell carcinoma (arrow). (b) The squamous cell carcinoma was seen as an iodine‐unstained area. (c) Endoscopic ultrasonography showed esophageal varix as dilated blood vessels in the submucosa on the anal side of the squamous cell carcinoma

**FIGURE 2:**
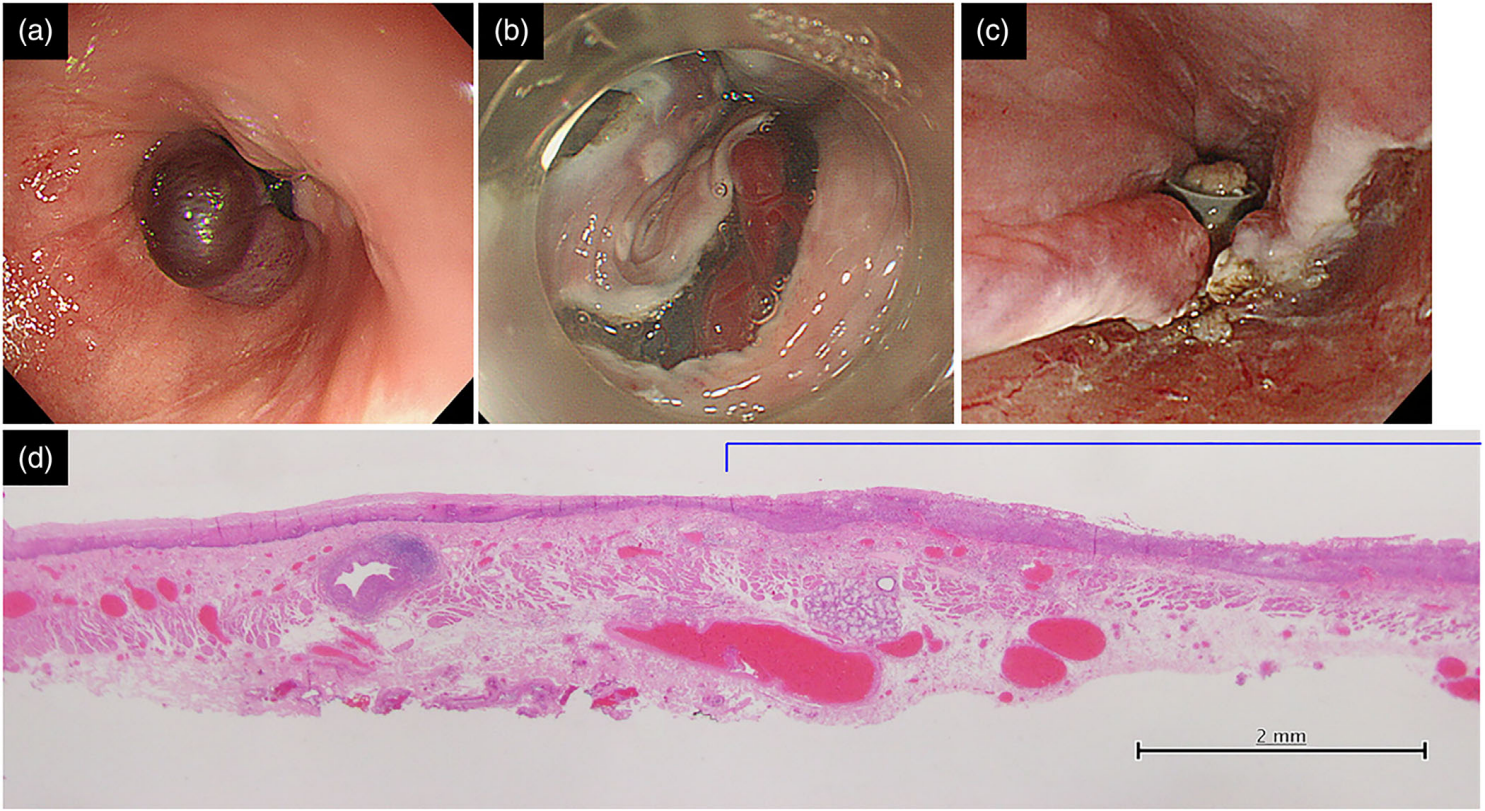
Images of case 1 during endoscopic treatment. (a) Endoscopic variceal ligation (EVL) was performed for esophageal varix. On the anal side of esophageal cancer, varix was ligated with two rings approximately 2 cm away from the squamous cell carcinoma. (b) Seven days after EVL, endoscopic submucosal dissection (ESD) was performed for squamous cell carcinoma. During the ESD procedure, dilated veins were found in the submucosa. (c) The EVL ring remained. The mucosal incision was possible on the oral side of the ring, and ESD was completed without any adverse events. (d) It is a loupe image in hematoxylin‐eosin staining of a resected specimen of ESD. The right side of the image is the oral side. As shown by the blue line, squamous cell carcinoma was seen in the mucosal epithelium. Dilated veins were found in the submucosa

Case 1: A 64‐year‐old man with alcoholic LC with Child‐Pugh classification A was found to have a SESCC on the site 36 cm from the incisor row by follow‐up EGD for a small EV that was detected 9 months earlier (Figure 1a,b). On endoscopic ultrasonography (EUS), a linear EV that was present on the anal side of the SESCC extended directly under the lesion (Figure 1c). We selected endoscopic variceal ligation (EVL) for EVs because the EV was not considered large enough for EIS with intravariceal injection of ethanolamine oleate (EO). To avoid EVL‐induced fibrosis of the submucosa, we placed an EVL ring 2 cm from the anal side of the SESCC (Figure 2a). Seven days after the EVL, we performed ESD under general anesthesia. After submucosal injection of sodium hyaluronate, we made an incision and dissection with a Dual knife and IT knife nano (Olympus Co., Tokyo, Japan) as high‐frequency knives. Although dilated blood vessels were seen in the submucosa, coagulation with hemostatic forceps could prevent bleeding (Figure 2b). Moreover, because of prominent submucosal fibrosis, we used scissors forceps (SB Knife Jr, Sumitomo Bakelite Co., Ltd., Tokyo, Japan) as needed. Finally, we successfully resected SESCC en bloc without significant bleeding (Figure 2c). The histopathological findings of the ESD specimens revealed a 15‐mm SESCC within the mucosal epithelium (Figure 2d). No adverse events occurred after ESD.

**FIGURE 3:**
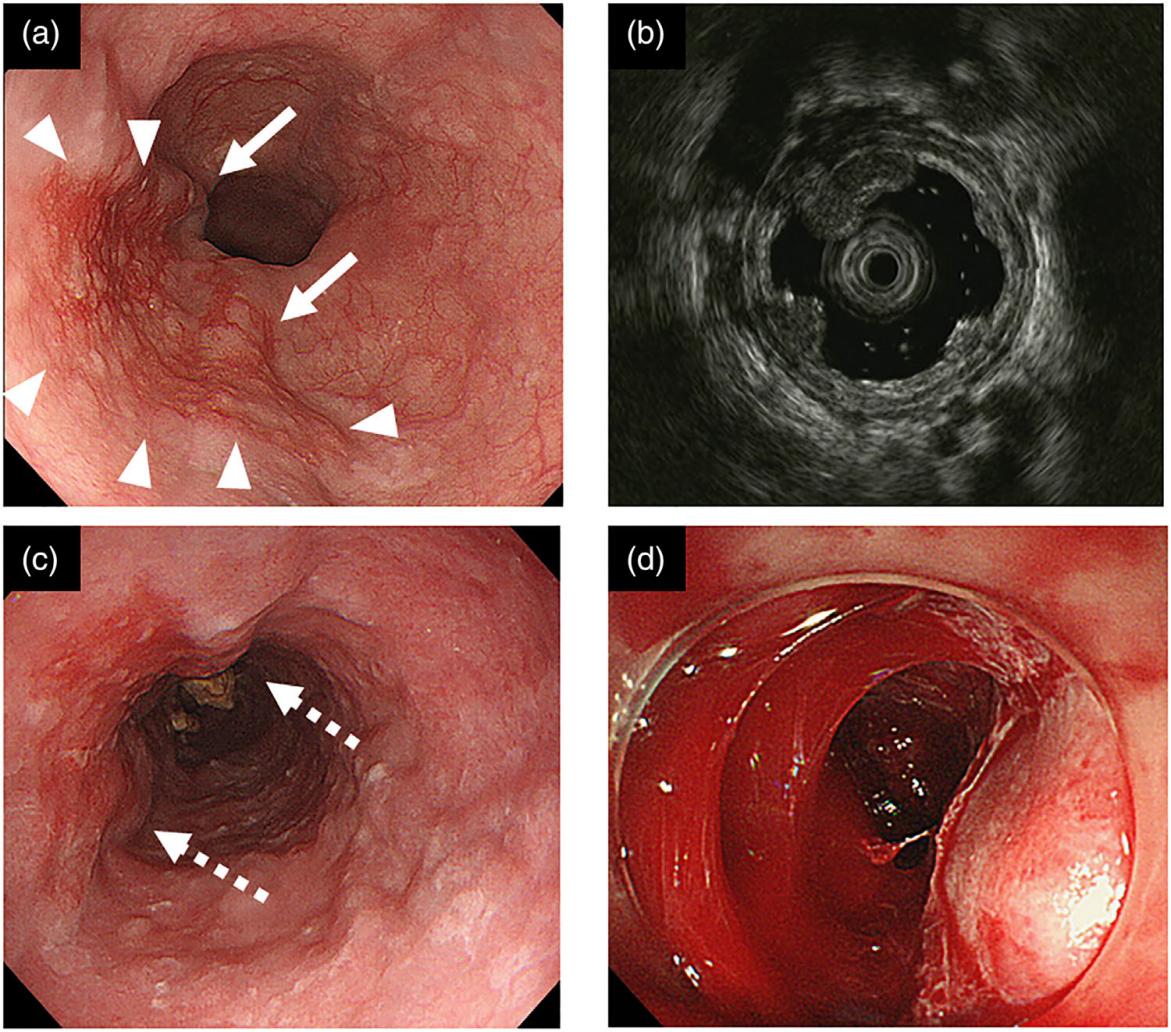
Endoscopic findings before treatment in case 2. (a) A flat, erythematous squamous cell carcinoma of 30 mm in size was detected on the left wall of the midthoracic esophagus (arrowhead). Two varices of F1 morphology were also observed (arrows). (b) Endoscopic ultrasonography (EUS) revealed varices in the submucosa just below the squamous cell carcinoma. (c) Two rings were applied to each of the two esophageal varices, taking care not to overlap the mucosal incision line on the antral side during endoscopic submucosal dissection (ESD). Seven days after the first EVL, the morphology of the esophageal varices remained (dashed arrows). (d) The bleeding could not be stopped during ESD, and ESD was discontinued

Case 2: A 59‐year‐old man with alcoholic LC with Child‐Pugh classification A was found to have a SESCC in the 33‐cm incisal row by screening EGD (Figure 3a). Two linear EVs were present on both sides of the SESCC. EUS showed the lumina of the EVs directly under the SESCC (Figure 3b). We prioritized EV treatment before ESD. We ligated two EVs on the anal side of the SESCC with two rings (total four rings). Seven days after the first EVL, we added EVL on the anal side of the SESCC, followed by EIS with extravariceal injection of polidocanol (2 ml), because the EVs remained (Figure 3c). Fourteen days after the first EVL, we administered 10 ml of polidocanol again, to regress still remaining dilated vessels detected on EUS. We considered that further EV treatment might cause strong fibrosis in the submucosa under the SESCC and make it difficult to perform planned ESD. Six days after the last EV treatment, we performed ESD under general anesthesia. Severe bleeding from the needle hole occurred after submucosal injection, and frequent bleeding also occurred during mucosal incision and submucosal dissection, requiring hemostatic forceps to stop the bleeding each time (Figure 3d). We also injected polidocanol into the submucosa to stop the bleeding. However, continuing the ESD became difficult due to insufficient submucosal endoscopic view, and we gave up. On EGD at 92 days after ESD, we ablated remnants of SESCC with argon plasma coagulation (APC). The EGD after 3 years of APC did not show recurrence of SESCC and EV bleeding.

## DISCUSSION

We report two cases of ESD performed on SESCC in patients with LC complicated by EVs. To the best of our knowledge, 30 SESCCs with EVs including two cases in our study have been reported to have undergone ESD (Table 1).^3^, ^4^, ^5^, ^6^, ^7^, ^8^, ^9^

**TABLE 1:** Cases of endoscopic submucosal dissection of esophageal cancer complicated with esophageal varices including our case

Author	Age	Sex	Cause of cirrhosis	Child‐Pugh classification	EV form	EV treatment	Interval between EV treatment and ESD	En bloc resection
Mitsuishi et al.^3^	59	M	Alcohol	A	F2	EIS (EO)	1 month	Yes
Mitsuishi et al.^3^	47	M	Alcohol	A	F2	EIS (EO)	1 month	Yes
Nishi et al.^†^	60	M	Alcohol	C	F1	EVL	19 days	Yes
Sawaguchi et al.^4^	55	N/A	Alcohol	A	F1	EVL	N/A	Yes
Sawaguchi et al.^4^	55	N/A	Alcohol	A	F1	EVL	N/A	Yes
Sawaguchi et al.^4^	67	N/A	Alcohol	A	F1	None	No EV treatment	Yes
Sawaguchi et al.^4^	67	N/A	Alcohol	A	F1	None	No EV treatment	Yes
Sawaguchi et al.^4^	70	N/A	HCV	C	F1	None	No EV treatment	Yes
Sawaguchi et al.^4^	70	N/A	Alcohol	A	F1	None	No EV treatment	Yes
Sawaguchi et al.^4^	68	N/A	Alcohol	A	F2	None	No EV treatment	Yes
Sawaguchi et al.^4^	69	N/A	Alcohol	A	F1	EVL	N/A	Yes
Sawaguchi et al.^4^	73	N/A	Alcohol	A	F1	None	No EV treatment	Yes
Hsu et al.^5^	52	M	Alcohol	N/A	N/A	EVL	N/A	Yes
Jovani et al.^6^	47	M	Alcohol	N/A	F2	EVL	N/A	Yes
Kinoshita et al.^7^	70	M	Alcohol	B	F2	EIS (EO)	1 month	Yes
Tsuo et al.^8^	N/A	M	N/A	A	F1	EVL	On the day	Yes
Tsuo et al.^8^	N/A	M	N/A	A	F1	EVL	On the day	Yes
Tsuo et al.^8^	N/A	M	N/A	A	F1	EVL	On the day	No (piecemeal resection)
Tsuo et al.^8^	N/A	M	N/A	A	F1	EVL	On the day	Yes
Tsuo et al.^8^	N/A	M	N/A	B	F2	EVL	On the day	Yes
Tsuo et al.^8^	N/A	M	N/A	B	F1	None	No EV treatment	No (failure*)
Fujimoto et al.^‡^	66	F	Alcohol	A	F1	EVL	2 months	Yes
Shiratori et al.^§^	70	M	N/A	N/A	F1	EVL	7 days	Yes
Xu et al.^9^	66	N/A	HBV	A	F1	None	No EV treatment	Yes
Xu et al.^9^	56	N/A	HBV	B	F1	None	No EV treatment	Yes
Xu et al.^9^	48	N/A	Alcohol	B	F2	EVL	1 month	Yes
Xu et al.^9^	66	N/A	Alcohol	C	F1	EVL	1 month	Yes
Xu et al.^9^	53	N/A	Alcohol	A	F2	TIPS	1 month	Yes
Present case 1	64	M	Alcohol	A	F1	EVL	7 days	Yes
Present case 2	59	M	Alcohol	A	F1	EVL/EIS (AS)	6 days	No (failure*)

Abbreviations: AS, aethoxysklerol; EIS, endoscopic injection sclerotherapy; EO, ethanolamine oleate; EVL, endoscopic variceal ligation; F, female; M, male; N/A, not available.

^†^ Nishi T, Toriumi F, Iwasaki E *et al. Prog Dig Endosc* 2014; **84**: 76–‐7.

^‡^ Fujimoto R, Shiozawa H, Nishina R *et al. Prog Dig Endosc* 2017; **90**: 80–‐1.

^§^ Shiratori Y, Ikeya T, Nakamura K. *ACG Case Rep J* 2019; **6**: e00185

* “Failure” means that ESD could not be completed.

Among those, the cause of LC was alcohol in 20 of 23 cases, and the Child‐Pugh classification was Grade A in 19, Grade B in 5, and Grade C in 3 cases. The morphology of EVs was F1 (liner) in 21 cases, F2 (beaded) in 8 cases, and F3 (tumorous) in none of the cases. Twenty‐one patients (70%) had prior treatment including EVL in 17 cases, EIS in 3 cases, and transjugular intrahepatic portosystemic shunt (TIPS) in 1 case. The en bloc resection rate by ESD was 90.0% (27/30).

Tsou et al.^8^ reported two unsuccessful cases of SESCC complicated by EV. In one case, piecemeal resection was performed due to inexperience of the surgeon's technique in the early stage of ESD introduction. In the other case, EV treatment was not performed before ESD because the patient had a circumferential SESCC of the lower esophagus and EVL had been performed repeatedly in the past. Consequently, ESD was stopped due to difficulty in dissection by the scaring and bleeding requiring blood transfusion.

In our unsuccessful case (case 2), EV was insufficiently treated by EVL and EIS with extravariceal injection before ESD, and the blood flow of the EV just below the SESCC remained on EUS images. Dilated vessels just below the SESCC, which could not be detected by endoscopic images alone, were observed. Although blood flow to these vessels should be blocked before ESD, we considered that treatment to eliminate the EVs could pose a risk of severe fibrosis of the submucosa and the difficulty of performing ESD. Therefore, despite the remaining EV blood flow, we proceeded to ESD; consequently, uncontrolled bleeding prevented the completion of ESD. Whether the bleeding was from the EV itself or from portal hypertension‐related dilated vessels was not identified. The left lateral recumbent position during ESD may have influenced the insufficient intraoperative visual field.

On the contrary, in case 1, ESD was completed without severe bleeding despite a mild scar in the submucosa due to EVL. In both cases 1 and 2, the morphology of the EVs was F1 and the Child‐Pugh classification was Grade A. On the other hand, case 1 had one EV under the SESCC, whereas case 2 had two EVs, which may have presented complex hemodynamics with the two EVs connected like a chain. As mentioned above, both cases underwent ESD in the left lateral recumbent position, but the SESCC in case 1 was above gravity while the SESCC in case 2 was below gravity.

EIS using intravariceal injection of EO before ESD has been reported to be less likely to cause submucosal fibrosis than EVL if EO does not leak from the EVs to the surrounding area.^3^, ^7^ We usually perform EIS for EVs, but in these two cases, we chose EVL because we considered injecting EO reliably into the EVs difficult. Although blood flow could not be blocked by EVL in case 2, the SESCC was finally eliminated by APC after an unsuccessful ESD. Tahara et al.^10^ reported that APC was performed on 21 lesions in 17 patients with SESCC who could not undergo ER because of severe comorbidities including LC. Two patients (9.5%) had SESCC remnants; however, additional APC was performed without recurrence. Therefore, APC may be an option for the treatment of SESCC cases associated with EV. Additionally, TIPS may also be effective as EV treatment that does not affect ESD of SESCC.^9^

In conclusion, we experienced two cases of SESCC with concomitant EV, in one of which ESD was discontinued due to uncontrolled intraoperative bleeding associated with EV. Scarring after EV treatment may make ESD procedures difficult. Therefore, ESD for SESCC with concurrent EV should be performed at a facility specializing in EV treatment, and other options such as APC or TIPS should also be considered.

## CONFLICT OF INTEREST

None.

## FUNDING INFORMATION

None.
